# Erratum to: The Wisconsin Hydrocephalus Survey: shunt-dependent hydrocephalus management style among members of the American Society of Pediatric Neurosurgeons

**DOI:** 10.1186/s12987-017-0069-y

**Published:** 2017-08-04

**Authors:** Mark Richard Kraemer, Bermans J. Iskandar

**Affiliations:** Department of Neurological Surgery, University of Wisconsin School of Medicine and Public Health, Madison, USA

## Erratum to: Fluids and Barriers of the CNS 2015, Volume 12 Suppl 1 DOI 10.1186/2045-8118-12-S1-O12

This abstract was unintentionally published twice in this supplement [1], by the same authors. Following should be considered the version of record and used for citation purposes: “Mark Richard Kraemer and Bermans J. Iskandar, The Wisconsin Hydrocephalus Survey: shunt-dependent hydrocephalus management style among members of the American Society of Pediatric Neurosurgeons, Fluids and Barriers of the CNS, Volume 12, Supplement Issue 1, 10.1186/2045-8118-12-S1-O12”. The duplicate “Mark Richard Kraemer and Bermans J. Iskandar, The Wisconsin Hydrocephalus Survey: shunt-dependent hydrocephalus management style among members of the American Society of Pediatric Neurosurgeons, Fluids and Barriers of the CNS, Volume 12, Supplement Issue 1, 10.1186/2045-8118-12-S1-O35” is to be ignored. Springer apologizes to the readers of the journal for not detecting the duplication during the publication process.
